# Voluntary Resistance Running as a Model to Induce mTOR Activation in Mouse Skeletal Muscle

**DOI:** 10.3389/fphys.2019.01271

**Published:** 2019-10-04

**Authors:** Gommaar D’Hulst, Andrew S. Palmer, Evi Masschelein, Ori Bar-Nur, Katrien De Bock

**Affiliations:** ^1^Laboratory of Exercise and Health, Department of Health Sciences and Technology, Swiss Federal Institute of Technology (ETH) Zurich, Zurich, Switzerland; ^2^Laboratory of Regenerative and Movement Biology, Department of Health Sciences and Technology, Swiss Federal Institute of Technology (ETH) Zurich, Zurich, Switzerland

**Keywords:** mTORC1, signaling, resistance running, load, acute

## Abstract

Long-term voluntary resistance running has been shown to be a valid model to induce muscle growth in rodents. Moreover, the mammalian target of rapamycin complex 1 (mTORC1) is a key signaling complex regulating exercise/nutrient-induced alterations in muscle protein synthesis. How acute resistance running affects mTORC1 signaling in muscle and if resistance applied to the wheel can modulate mTORC1 activation has not yet been fully elucidated. Here, we show that both acute resistance running and acute free running activated mTORC1 signaling in the *m. gastrocnemius, m. soleus, and m. plantaris*, but not in *m. tibialis anterior* of mice when compared to sedentary controls. Furthermore, only the low threshold oxidative part in the m. gastrocnemius showed increased mTORC1 signaling upon running and acute heavy-load resistance running evoked higher downstream mTORC1 signaling in both m. soleus and m. plantaris than free running without resistance, pointing toward mechanical load as an important independent regulator of mTORC1. Collectively, in this study, we show that voluntary resistance running is an easy-to-use, time-efficient and low stress model to study acute alterations in mTORC1 signaling upon high-load muscular contractions in mice.

## Introduction

Maintaining skeletal muscle mass throughout life is critical as loss of muscle mass is associated with increased mortality (Szulc et al., 2010), higher disability, loss of function (Janssen et al., 2002), and increased risk of falls (Szulc et al., 2004). Resistance exercise increases muscle mass (Bhasin et al., 1996; Abe et al., 2003; Verdijk et al., 2009) and improves strength outcomes (Pyka et al., 1994; Staron et al., 1994), but the mechanisms by which high load contractions regulate skeletal muscle fiber size are incompletely understood. In an attempt to elucidate these mechanisms, many resistance-based exercise models in mice have been developed (Cholewa et al., 2014), such as synergistic ablation (Goldberg, 1968), electrical stimulation (Baar and Esser, 1999), and chronic stretch (Goldspink, 1999). Although all valuable, they are invasive, cumbersome, and often do not mimic real life scenarios. Remarkably, many mouse strains voluntarily run large distances when given access to a running wheel (De Bono et al., 2005). Moreover, external resistance can be added to the wheel to increase muscle force production (Soffe et al., 2016). Therefore, as mouse handling and stress is minimal, the running wheel is considered an excellent model of physiological training with similar muscular adaptations as other well-accepted models such as synergistic ablation and electrical stimulation. Indeed, many groups have shown that voluntary resistance running leads to muscular hypertrophy in rodents (Legerlotz et al., 2008; Call et al., 2010; White et al., 2016; Mobley et al., 2018).

mTOR is a protein complex, which acts as a conductor in cell growth (Wolfson and Sabatini, 2017). In particular, mTOR complex 1 (mTORC1) integrates nutrient, growth, and stress signals to promote protein synthesis (Goodman et al., 2011; Huang and Fingar, 2014). Activation of mTOR is known to increase ribosomal biogenesis and protein translation (Mahajan, 1994; Thoreen et al., 2012; Figueiredo et al., 2015), two processes, which play a central role in the adaptation to resistance training or chronic overload (Bodine et al., 2001; Drummond et al., 2009; Ogasawara and Suginohara, 2018). Both a single bout or repeated bouts of resistance exercise can increase mTOR signaling in muscle (Baar and Esser, 1999; Kubica et al., 2005; Burd et al., 2010; Dreyer et al., 2010) and an enhancement in mTOR activation after resistance exercise is related to increased muscle mass (Baar and Esser, 1999). To confirm the role of mTOR on muscle hypertrophy, blocking mTOR signaling with compounds such as rapamycin or AZD8055 has been demonstrated to reduce protein synthesis and muscle growth (Bodine et al., 2001; Drummond et al., 2009; Ogasawara and Suginohara, 2018), underscoring the indispensable role of mTORC1 in muscular adaptations to resistance type exercise. Data on voluntary resistance running and mTOR activation are sparse, but one study showed no increase in mTOR activation after 8 months of resistance running in the m. quadriceps, despite hypertrophy in most of the hindlimb muscles, including the m. quadriceps (White et al., 2016). Interpretation of this data might be difficult since the mice were aged, samples were harvested several hours after exercise, no standardization of food intake before sample harvesting occurred, and – consistent with human data (Brook et al., 2015) – resistance training could have resulted in an attenuated activation of mTORC1 in response to resistance training. Additionally, other hindlimb muscles were not examined for downstream mTORC1 signaling (White et al., 2016). Thus, to date, the mechanisms behind resistance running-induced muscle hypertrophy are unclear.

Therefore, the aim of this study was to explore whether voluntary resistance running can be used as a non-invasive stress-free model to study and acutely modulate mTORC1 signaling *in vivo*.

## Materials and Methods

### Animals

All experiments were performed on male C57BL/6 J mice. All mice used for the experiments were housed in individually ventilated cages (3–4 littermates per cage) at standard housing conditions (22°C, 12 h light/dark cycle, dark phase starting at 7 pm), with ad libitum access to chow (KlibaNafag, diet #3436 and diet #3437) and water. Health status of all mouse lines was regularly monitored according to FELASA guidelines.

### Experimental Procedures

All animal procedures were approved by the Veterinary office of the Canton of Zürich (license nr. ZH254-16). During the intervention, mice were individually housed in open cages equipped with a running wheel device (TSE Systems). The running wheel device continuously records wheel movements out of which total distance (km), speed (m.s^−1^), number of running bouts, and resistance on the wheel (N) can be extracted. Additionally, to increase the force needed to rotate the wheel, resistance (0–100%) can be added. To calculate total work, we used ***W* = *Pt***, where *W* is work, *P* is power, and *t* is time. To calculate the power of the wheel at each braking resistance, we used the equation ***P* = 2π × *f* × *M***, where *f* is the angular frequency of the wheel, and *M* is the torque at a given braking resistance. TSE Systems provided a torque braking resistance curve for which a braking resistance of 60% has a constant torque of 0.0125 N.m.

#### Experiment 1

**Figure 1:**
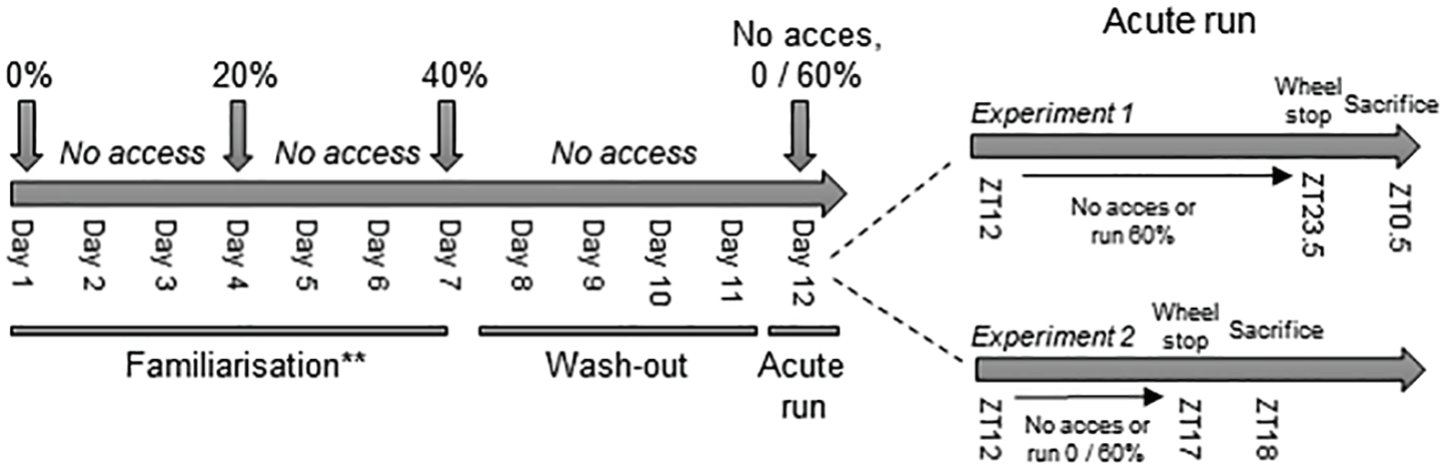
Experimental set-up. ** In experiment 1, only the VResRun group was familiarized to the wheel. The Sed remained sedentary during the whole experiment. In experiment 2, all groups were familiarized to the wheel, including the Sed.

An overview of the experimental procedures can be found in Figure 1. About 14 to 16-week-old male C57BL/6 J mice were randomly assigned to either the voluntary resistance-running group (VResRun) or sedentary (Sed) control group. VResRun mice were familiarized to the resistance running protocol for 1 week. During familiarization, VResRun mice had access to the wheel; with 0% braking resistance on the first night, with 20% braking resistance on the fourth night, and with 40% braking resistance on the seventh night. On the second, third, fifth, and sixth night, the running wheels were blocked. After the seventh night, mice remained in the resistance wheel cages without access to the running wheel for four nights to mitigate any potential training effects. On the twelfth and final day of the intervention, mice had access to the running wheel at 60% braking resistance for the 12-h dark cycle (ZT12-ZT23.5). Sedentary mice did not undergo familiarization and were housed in a resistance running wheel cage with the wheel blocked. At ZT23.5, running wheels were blocked, mice were removed from running cages and fasted for 1 h. Muscle samples were collected 1 h after cessation of running, since at this time point, eccentric contraction induced activation of mTORC1 is maximal (O’Neil et al., 2009).

#### Experiment 2

Around 10–16-week-old male C57BL/6 mice were randomly allocated either to a Sed, voluntary run (VRun) or VResRun group. This time, Sed, VRun, and VResRun groups were all familiarized following the aforementioned familiarization protocol (Figure 1). Access to the running wheels was blocked for four nights. On the final night, VResRun mice ran at 60% braking resistance, Run mice ran at 0% braking resistance from ZT12 to ZT17 and Sed did not run (wheel blocked). Mice were removed from running cages at ZT17 am and fasted for 1 h prior to sample collection.

### Sample Collection

Mice were fasted for 1 h at ZT23.5 (experiment 1) and ZT17 (experiment 2) and anesthetized using Ketamine/Xylazine 10 μg.g^−1^ and acepromazine (2–5 mg.kg^−1^) body weight *via* intraperitoneal injection. The depth of anesthesia was confirmed by testing pedal withdrawal reflex before tissue collection. Subsequently, the *m. gastrocnemius (GAS), m. tibialis anterior (TA), m. soleus (SOL), m. plantaris (PLT)* and *m. triceps (TRI)* were dissected and snap frozen. One GAS was frozen in OCT embedding matrix (CellPath) in 2-Methylbutane (sigma Aldrich) on liquid nitrogen for histochemical analysis. After sample collection, animals were euthanized and major bleeding was induced to confirm death.

### Protein Extraction and Western Blot

#### Sample Preparation

Between 10 and 25 mg of muscle sample was homogenized in ice cold lysis buffer (1:10, w/v) (50 mM Tris-HCl pH 7.0, 270 mM sucrose, 5 mM EGTA, 1 mM EDTA, 1 mM sodium orthovanadate, 50 mM glycerophosphate, 5 mM sodium pyrophosphate, 50 mM sodium fluoride, 1 mM DTT, 0.1% Triton-X 100 and 10% protease inhibitor) (20 μl per 1.8–2.5 of tissue sample) using an OMNI-THq Tissue homogenizer (OMNI International) for 20 s until a consistent homogenate was formed. Samples were centrifuged at 4°C at 10,000 g for 10 min and the supernatant with proteins collected. Protein concentration was determined using the DC assay protein method (Biorad Laboratories) to equalize the amount of protein.

#### Protein Transfer

Samples were prepared 3:4 with laemmli buffer containing 10% 2**-**mercaptoethanol (Bio-rad laboratories) and heated at 95° for 5 min. Proteins were run on a 4–20% Mini-PROTEAN TGX Stain Free Pre-Cast Gel (Biorad Laboratories) for 45 min at 120–140 V and subsequently transferred onto immuno-Blot PVDF Membranes (Biorad Laboratories) at 90 V for 100 min. Membranes were cut according to desired proteins, blocked for 1 h at room temperature in 5% milk in TBS-T, TBS (1:10, w/v) (24.23 g Trizma HCl, 80.06 g NaCl in 800 ml ultra-pure water pH 7.6, topped up to 1 L) with 1 ml of Tween, and incubated overnight with the following primary antibodies 1:1,000 (Cell Signaling); pS6K1^Thr389^ (#9206), pS6K1^Thr421/Ser424^ (#9204), pRPS6^Ser235/236^ (#2211), pSMAD2^Ser245/250/255^ (#3104), p4E-BP1Ser^65^ (#9451), and pSAPK/JNK^Thr183/Tyr185^ (#9251). The membranes were washed for 10 min three times in TBS-T and subsequently incubated in secondary antibody 1:5,000 in TBS-T with 5% milk (Anti-rabbit IgG, HRP-linked Antibody #7074, Cell Signaling) for 1 h at room temperature. Proteins were washed for 10 min three times in TBS-T and incubated for 30 s in 1:1 Luminol/Enhancer solution and peroxide solution. Membranes were imaged with a Biorad Chemidoc Touch Imaging System (Biorad Laboratories).

#### Image Analysis

Images were quantified using Image Lab software (Biorad Laboratories) using the volume tools function with each band and lane quantified with the same volume or area. Total membrane protein or total gel loading was used as a loading control (Rivero-Gutiérrez et al., 2014) and a positive control sample was loaded to compare across gels. All quantified volumes were divided by the positive control and normalized to the control group.

### Immunohistochemistry

#### Sample Preparation

##### P-RPS6

About 10 μm sections of muscle embedded in OCT were made using a cryostat (Leica CM 1950) and collected on Superfrost Ultra Plus slides (Thermo Scientific). Muscle samples were fixed with −20°C acetone for 10 min and subsequently incubated in PBS for 15 min. Slides were blocked for 1 h at room temperature in solution A (PBS with 5% normal goat serum and 0.3% CHAPS) and then incubated overnight at 4°C in solution B (PBS with 0.5% BSA and 0.3% CHAPS) containing primary antibody p-RPS6^Ser235/236^ rabbit conjugated (1:200, cell signaling). The next day samples were washed three times with PBS and then incubated for 1 h at room temperature in solution A containing secondary antibodies Goat-anti rabbit IgG Alexa Fluor 488 (1:250, Invitrogen). Slides were once again washed two times for 5 min in PBS and then washed for 10 min with PBS containing WGA alexa fluor 647 (1:400, Invitrogen). Slides were mounted with immuno-mount (Thermo Scientific) and a glass coverslip and allowed to dry. A sample that followed the above procedure but was not incubated with primary antibodies was used as a negative control. Images were taken using an epifluorescence microscope (Zeiss Axio observer Z.1) at 20× using Zen Pro software. An image of a sedentary sample was used to define imaging settings and applied to all subsequent images.

#### Fiber Typing

About 10 μm sections were dried and washed for 5 min in PBS supplemented with 0.05% triton (PBST) and subsequently blocked for 60 min in PBST +10% goat serum (ThermoFisher Scientific, 16200-064). Afterward, a primary antibody cocktail was applied for 120 min for myosin heavy chain I (1/50), IIa (1:200) (Developmental studies hybridoma bank) diluted in PBST +10% goat serum. After washing three times for 5 min, a secondary antibody cocktail, diluted in PBST +10% goat serum, was applied for goat anti-mouse Alexa Fluor 488, 350 and wheat germ agglutinin Alexa fluor 647 (1:250) for 60 min. Slides were mounted after a 3 × 5 min wash, sealed with glass cover slips and imaged with a epifluorescent microscope (Zeiss Axio observer Z.1) at 10×.

### Statistical and Data Analyses

Results are presented as mean with standard error of the mean (SEM) bars and individual data points. An unpaired two-tailed Student’s *t*-test was used for generating a *p* in experiment 1 when comparing two groups. Data in experiment 2 were subjected to a one-way analysis of variance (ANOVA) to generate a *p* value and *post hoc* tests were performed using Tukey’s *post hoc* test using Graphpad Prism to compare between groups. Significance was set at *p* < 0.05.

## Results

### Acute Resistance Running Activates the mTOR Signaling Pathway in Mouse Skeletal Muscle

**Figure 2:**
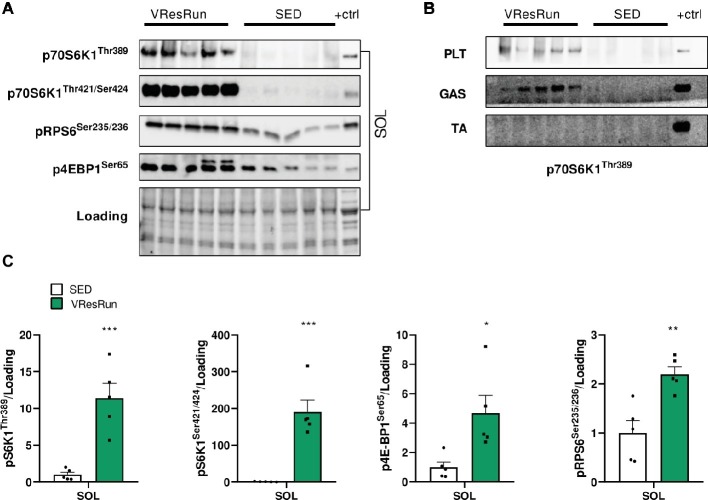
The effect of one night of voluntary resistance running (VResRun, *n* = 5) on mTORC1 downstream signaling. **(A)** Representative blots of pS6K1^Thr389^, pS6K1^Thr421/Ser424^, pRPS6^Ser256/236^, p4EBP1^Ser65^ in SOL. **(B)** Representative blots of pS6K1^Thr389^ in PLT, GAS, and TA. **(C)** Quantifications. (Sed; *n* = 5). *p < 0.05 vs. SED, **p < 0.01 vs. SED, ***p < 0.001 vs. SED.

To determine if resistance exercise activates mTOR signaling within skeletal muscle, we measured the phosphorylation of downstream mTORC1 target kinases 1 h after cessation of one night of resistance running. In mice, phosphorylation levels of pS6K1^Thr389^, pS6K1^Thr421/Ser424^, pRPS6^ser235/236^, and p4EBP1^ser65^ were highly increased in SOL of VResRun compared to SED mice (Figures 2A,C). Furthermore, VResRun also lead to a strong increase pS6K1^Thr389^ in PLT and GAS (Figure 2B). Interestingly, there were no significant differences in phosphorylation of any mTOR targets in the TA muscle (Figure 2B) or TRI (data not shown). This data shows that VResRun activates downstream mTORC1 signaling.

**Figure 3:**
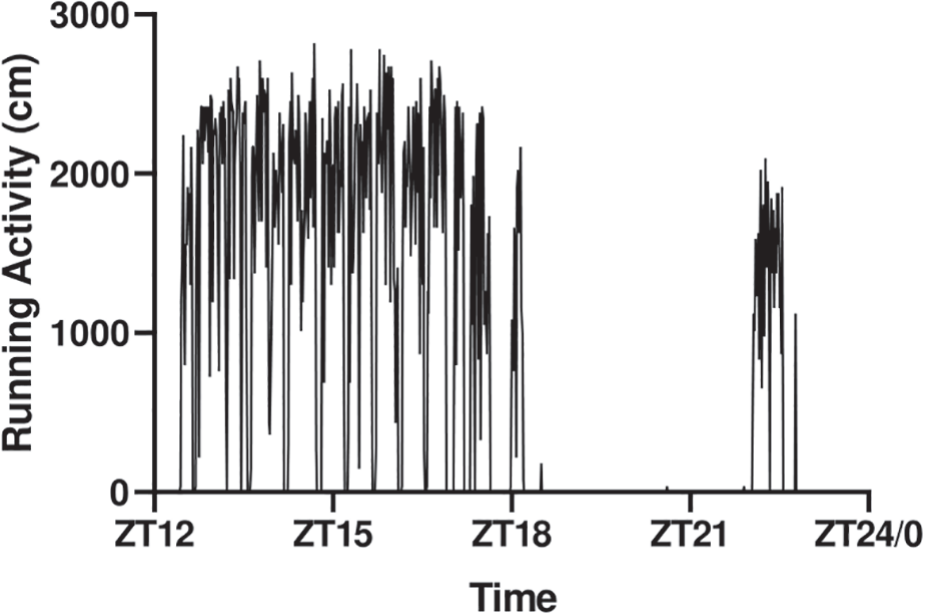
Representative running pattern of a mouse during the active phase.

The running activity over the night followed the pattern seen in Figure 3. Mice ran consistently and constantly between ZT12 and ZT18, after which they rested before recommencing running after ZT22. Interestingly, some mice did not restart at ZT22 for unknown reasons, a feature that could potentially induce variability in the signaling data. We therefore decided to further standardize the protocol by blocking the wheel at ZT17 and excise the muscles at ZT18 in experiment 2.

### Acute Resistance Running Activates Mammalian Target of Rapamycin Complex 1 More Than Non-resistance Running

**Figure 4:**
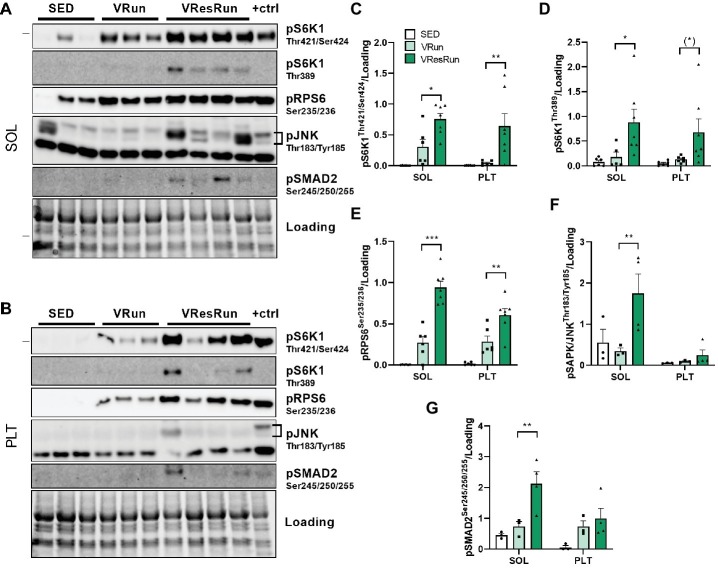
The effect of 5 h of voluntary running (VRun, *n* = 6) and voluntary resistance running (VResRun, *n* = 7) on mTORC1 downstream signaling. **(A)** Representative blots in SOL. **(B)** Representative blots in PLT. **(C)** Quantification of pS6K1^Thr421/Ser424^. **(D)** Quantification of pS6K1^Thr389^. **(E)** Quantification of pRPS6^Ser235/236^. **(F)** Quantification of pSAPK/JNK^Thr183/Tyr185^. **(G)** Quantification of pSMAD2^Ser245/250/255^. (Sed, *n* = 6). *p < 0.05 vs. SED, **p < 0.01 vs. SED, ***p < 0.001 vs. SED.

For the second experiment, we used the optimized protocol, and included an additional condition where no resistance was applied on the wheel (VRun). This would allow us to assess whether the observed increase in mTORC1 activity was caused by the increase in resistance applied on the wheel, rather than by running itself. Parameters from the free running and the resistance running bout are presented in Table 1. VResRun had to overcome 24 times more force per rotation of the wheel than the VRun, leading to 29 times more total work over the course of the 5 h run time-window (*p* < 0.001, Table 1). Both acute VRun and VResRun increased downstream mTORC1 signaling in SOL and PLT as measured by pS6K1^Thr421/Ser424^, pS6K1^Thr389^, and pRPS6^ser235/236^ (Figures 4A–E). Notably, VResRun augmented downstream mTORC1 signaling significantly more than VRun both in the SOL and PLT, indicating that load independently augments mTORC1 signaling in an *in vivo* setting (Figures 4A–E). Finally, total work was highly correlated with pS6K1^Thr421/Ser424^ in both SOL (*r* = 0.69, *p* < 0.05) and PLT (*r* = 0.63, *p* < 0.05).

**Table 1:** Running summary VRun vs. VResRun.

	VRun	VResRun
Distance (km)	3.46 ± 0.95	4.00 ± 0.40
Time (min)	129 ± 31	151 ± 12
Sum of all runs	1,014 ± 272	876 ± 278
Speed (m.s^−1^)	0.44 ± 0.05	0.44 ± 0.02
Work (J)	30 ± 8	867 ± 88^***^
Force (N)	0.009	0.22^***^

*** *Indicates p < 0.001 vs. VRun. VRun, n = 6; VResRun, n = 7*.

One of the proposed pathways of how high-load contractions regulate mTORC1 is *via* activation of the stress responsive mitogen-activated protein kinase (MAPK) pathway (Rahnert and Burkholder, 2013). More specifically, c-Jun N-terminal kinase (JNK), a member of the MAPK family, has been put forward as the molecular switch that, when active, promotes muscle hypertrophy and, when inhibited, allows endurance training adaptations such as enhanced aerobic capacity (Lessard et al., 2018). To investigate whether the increased mTORC1 signaling in VResRun muscle was related to increased JNK activation when compared to predominantly aerobic (VRun) or sedentary (SED) muscle, we measured pSAPK/JNK at Thr183/Tyr185 and its downstream target pSMAD2 at Ser245/250/255. VResRun increased pJNK and pSMAD2 modestly (~4 fold, *p* < 0.05) in SOL only, while PLT showed higher variation and was unaffected (Figures 4A,B,F,G). Thus, while we observed increased mTORC1 activation upon VRun, this seemed not to be dependent on increased JNK and SMAD2 phosphorylation (Figures 4A,B,F,G).

**Figure 5:**
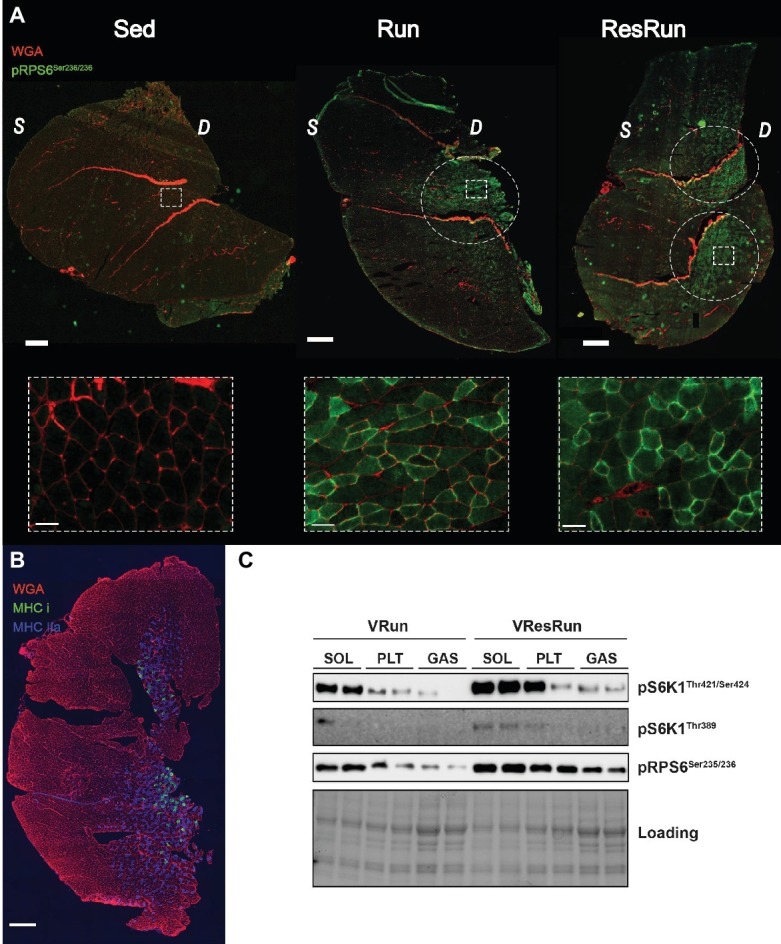
**(A)** Representative immunofluorescence pictures of GAS in Sed, VRun, and VResRun with pPRS6^Ser235/236^ in green and WGA in red. **(B)** Representative immunofluorescence staining of GAS with Myosin Heavy Chain type I in green, Myosin Heavy Chain type IIa in blue and Wheat Germ Agglutinin WGA in red. **(C)** Representative blots of downstream mTORC1 signaling (VRun and VResRun) in SOL, PLT, and GAS. Scale bar in tile scan represents 500 μm and scale bar in zoom-in represents 50 μm; WGA, wheat germ agglutinin; *S,* superficial part; *D,* deep part.

To visualize activation and distribution of mTORC1 in mouse skeletal muscle, we performed an immunohistochemical staining for phospho-RPS6^ser235/236^ in GAS, a muscle that consists of both oxidative and glycolytic fibers, organized in specific regions. Interestingly, only a selected amount of fibers were phosphorylated in the VRun and VResRun (Figure 5A) and they were exclusively situated in the oxidative part (on the anterior side) of the GAS (Figure 5B; Sher and Cardasis, 1976). This also represented itself in a lesser activation of downstream mTORC1 after acute running when the phosphorylation of mTORC1 was measured in the whole GAS compared to the SOL and PLT (Figure 5C). These data suggest that voluntary running activates downstream mTORC1 signaling in a select amount of predominantly oxidative fibers.

## Discussion

This study aimed to validate acute resistance running as a model of resistance exercise-induced mTORC1 activation in mouse skeletal muscle. Previous research has shown muscle hypertrophy (Legerlotz et al., 2008; White et al., 2016) and increased strength (Roemers et al., 2017) following voluntary resistance running, and we aimed to link these models with a key molecular pathway, which regulates muscle protein synthesis. We show increased mTORC1 activity in the PLT, SOL, and GAS, whereas we did not detect enhanced activation in the TA or TRI muscle, suggesting muscle specific differences in mTORC1 activation upon acute resistance running, which might be determined by specific load applied on these muscles during running. To further study the involvement of mechanical load, we subjected mice to voluntary free and resistance running and found increased downstream mTORC1 signaling in SOL and PLT when resistance was applied to the wheel.

Numerous studies showed increased muscle mass after resistance running in mice. It has been reported that muscle mass is increased in the PLT (Legerlotz et al., 2008; Holland et al., 2016; Mobley et al., 2018), SOL (Konhilas et al., 2005; Legerlotz et al., 2008; Call et al., 2010; White et al., 2016), GAS (Mobley et al., 2018), *m. quadriceps* (Soffe et al., 2016), and TRI (Call et al., 2010) after long term (>4 weeks) resistance running. Our findings provide evidence of a potential molecular basis for the above findings as we report increased mTORC1 activation after one night of resistance running in the aforementioned muscles. It has been reported before that during wheel running, the force shifts to the hindlimb (0.85:0.15) compared to overground running leading to a subsequent increase in hindlimb normal forces (Roach et al., 2012). It is therefore likely that the activation of mTORC1 after resistance running was load-dependent, as we did not find any, or minimal phosphorylation of its downstream kinases in the TRI of the forelimb. In agreement with this, we also failed to observe significant mTORC1 activation in TA, a dorsiflexor muscle that does not bear body weight, can produce less force than the plantar flexors (Ashton-Miller et al., 1992) and thus is likely recruited to a lower extent during (resistance) running when compared to muscles of the *m. triceps surae*.

At 60% resistance, the resistance we opted to put on the wheel in this study, the additional force needed to overcome the wheel resistance was 0.2 N. Plantar flexion strength of the *m. triceps surae* is much higher [3.3 N (Ashton-Miller et al., 1992)] indicating that maximal force production was not required to overcome the resistance imposed by the wheel. Thus, during a single contraction only a subset of motor units, likely the oxidative fibers which are recruited during submaximal force production (Henneman et al., 1965), needed to be activated. This was underscored by our observations that in the GAS, only the areas containing more oxidative fibers (the anterior part close to the bone) were highly positive for pRPS6, while the glycolytic part remained unaffected after VResRun and VRun. These findings are corroborated by the fact that nearly all aforementioned studies investigating hypertrophy after wheel running found significant hypertrophy in the SOL and/or PLT, while GAS and dorsiflexors were much less, or even not at all affected (Legerlotz et al., 2008; White et al., 2016). Nevertheless, future research needs to further evaluate whether load-dependent mTORC1-signaling directly affects hypertrophy in a muscle-specific way.

Besides to a large increase in downstream mTORC1 signaling after voluntary resistance running, we also observed a significant, albeit ~50% less potent increase in pS6K1 and pRPS6 in mice that acutely ran for a night without any resistance on the wheel. To the best of our knowledge, this is the first study that specifically alters load in an acute *in vivo* physiological training setting to modulate intra-muscular downstream mTORC1 signaling. The increase in mTORC1 activity after acute endurance exercise (VRun) was somewhat surprising as previous data has reported a decrease in mTORC1 activation 0.5, 3 and 6 h after a single bout of uphill treadmill running (18 m.min^−1^ for 1 h at a 5° gradient) (Philp et al., 2015). On the other hand, studies using longer duration (and intensity) of running did indeed find higher downstream mTORC1 signaling immediately and 3 h after the running bout (Scribbans et al., 2014). In an attempt to elucidate the mechanisms that regulate muscular plasticity, a recent report showed JNK/SMAD2 to act as a major switch between muscle growth and endurance phenotype when the kinases were activated or inactivated, respectively (Lessard et al., 2018). We report an increase in pJNK1/2 and pSMAD2 in VResRun, while this effect was absent in the VRun condition. The absence of JNK activation and the induction of mTORC1 after VRun suggest that VRun is indeed a good model for endurance adaptations in skeletal muscle and that mechanisms other than the mechanosensitive MAPK signaling regulate mTORC1 upon endurance type training.

To conclude, we show that acute resistance running and acute free running increased activation of the mTORC1 pathway in mouse muscle. Our data suggest that this activation is dependent on load as the *m. triceps surae* muscles had higher activation compared to the hindlimb dorsi flexors and only a subset of oxidative fibers were activated in the GAS. Furthermore, resistance running, in which the force per contraction is increased compared to free wheel running, induced a more robust mTORC1 activation in both SOL and PLT. In this work, we present an easy-to-use, low stress, and time-efficient model to study mTORC1 signaling after resistance training in mice, which can be used in future studies to pin-down the molecular signaling events that determine muscle growth upon muscular contractions.

## Data Availability Statement

The datasets generated for this study are available on request to the corresponding author.

## Ethics Statement

All animal procedures were approved by the Veterinary office of the Canton of Zürich (license nr. ZH254-16).

## Author Contributions

GD’H and AP designed the study, wrote the manuscript and performed all the experiments and data analysis. EM helped performing the experiments and edited the manuscript. OB-N helped in drafting and revising the manuscript. KB designed the study and helped drafting and revising the manuscript.

### Conflict of Interest

The authors declare that the research was conducted in the absence of any commercial or financial relationships that could be construed as a potential conflict of interest.
